# Effect of Sterilization Methods on the Physicochemical Properties of Silk Fibroin Hydrogels

**DOI:** 10.3390/polym18131625

**Published:** 2026-06-30

**Authors:** Carlos A. Busatto, Emanuela Callone, Marzia Di Chio, Sandra Dirè, Chavee Laomeephol, Ilaria Decimo, Adriano Fasolo, Stefano Ferrari, Erika Bonacci, Emilio Pedrotti, Antonella Motta

**Affiliations:** 1 BIOtech—Center for Biomedical Technologies, Department of Industrial Engineering, University of Trento, 38183 Trento, Italy; carlos.busatto@unitn.it; 2 Instituto de Desarrollo Tecnológico para la Industria Química (INTEC), Consejo Nacional de Investigaciones Científicas y Técnicas (CONICET), Universidad Nacional del Litoral (UNL), Santa Fe 3000, Argentina; 3 “Klaus Müller” Magnetic Resonance Laboratory, Department of Industrial Engineering, University of Trento, 38123 Trento, Italy; emanuela.callone@unitn.it (E.C.); sandra.dire@unitn.it (S.D.); 4 Ophthalmic Unit, Department of Neurosciences, Biomedicine and Movement Sciences, University of Verona, 37134 Verona, Italy; marzia.dichio@univr.it (M.D.C.); ilaria.decimo@univr.it (I.D.); emilio.pedrotti@univr.it (E.P.); 5 Department of Biochemistry and Microbiology, Faculty of Pharmaceutical Sciences, Chulalongkorn University, Bangkok 10330, Thailand; chavee.l@chula.ac.th; 6 Fondazione Banca degli Occhi del Veneto, 30173 Venice, Italy; adriano.fasolo@fbov.it (A.F.); stefano.ferrari@fbov.it (S.F.); 7 Department of Translational Medicine, University of Ferrara, 44121 Ferrara, Italy; 8 Department of Engineering for Innovation Medicine, Ophthalmology Clinic, University of Verona, 37134 Verona, Italy; erika.bonacci@univr.it

**Keywords:** methacrylated silk fibroin, hydrogels, sterilization, physicochemical properties, tissue engineering

## Abstract

This study investigates the impact of clinically relevant sterilization methods, such as ethylene oxide (EtO), gamma (γ) irradiation, autoclaving, and hydrogen peroxide gas plasma, on the physical, structural, and functional properties of methacrylated silk fibroin hydrogels obtained by photo- and enzymatic crosslinking. EtO, γ irradiation, and hydrogen peroxide gas plasma were applied as terminal sterilization methods to the fabricated hydrogels, whereas autoclaving was performed on the SilMA precursor solution prior to hydrogel formation. Silk fibroin hydrogels at 5 and 7 wt.% concentrations were evaluated for transparency, rheological behavior, water content, secondary structure, chemical composition, thermal stability, microbial growth, and morphology after sterilization and during storage. EtO sterilization effectively maintained high optical transparency (>98%) but compromised the mechanical properties of the hydrogels. In contrast, γ-irradiation and autoclaving promoted greater β-sheet formation, resulting in increased mechanical stiffness and thermal stability but reduced transparency after autoclaving. Plasma sterilization proved unsuitable, as incomplete cycles related to the high-water content of hydrogels. Overall, the results delineate the influence of different sterilization strategies and identify approaches that best preserve or enhance the performance of silk hydrogels, supporting their clinical translation in tissue engineering.

## 1. Introduction

Tissue engineering is an interdisciplinary field including experts from biology, engineering, and material science to develop functional constructs that restore, maintain, or improve damaged tissues or organs [1]. Silk fibroin, a fibrous protein derived from silkworm cocoons, has gained particular attention as a biomaterial in regenerative medicine due to its exceptional properties, including mechanical strength, biocompatibility, low immunogenicity, biodegradability [2,3], and the ability to be processed into various formats such as films, sponges, nanoparticles, and hydrogels [4,5,6]. In addition, its capacity to support cell adhesion, proliferation, and differentiation makes it particularly interesting for tissue engineering applications [7,8].

Silk fibroin scaffolds are typically derived from aqueous silk fibroin solutions prepared through different multistep protocols, allowing the final material properties to be tailored to specific application requirements [9]. The structural and mechanical properties of the resulting biomaterials are influenced by several factors, including the characteristics of the biopolymer itself, extraction conditions (particularly degumming time), processing conditions, and post-processing parameters like the sterilization technique and the extent of β-sheet formation [6,10,11].

Silk fibroin–based hydrogel formulations represent a particularly promising class of biomaterials for reproducing the structural and mechanical properties of native tissues. These systems provide a hydrated three-dimensional matrix capable of closely mimicking the extracellular environment and have shown considerable potential across multiple biomedical applications, including cartilage regeneration [12], skin repair [13], and ocular tissue engineering [14,15]. Within the field of corneal regeneration, silk fibroin hydrogels are especially attractive due to their intrinsic optical transparency, favorable mechanical performance, and capacity to support epithelial cell proliferation, all of which are critical for functional restoration of the cornea following injury or disease [7,16].

Despite these advantages, the clinical translation of silk-based materials faces several challenges, among which sterilization remains a crucial issue that needs to be carefully addressed. The sterilization process must effectively eliminate microbial contamination while preserving the material’s functional integrity [17]. A variety of sterilization techniques have been investigated for silk fibroin-based biomaterials, each exerting distinct effects on the protein’s molecular structure and resulting material properties [18,19].

Previous studies have evaluated several commonly used sterilization methods, including autoclaving (moist heat), γ-irradiation, dry heat, ethanol immersion, EtO exposure, and H_2_O_2_ gas plasma treatment. In this context, Hofmann et al. (2014) [18] investigated the effects of different sterilization methods on the properties of porous silk fibroin scaffolds. Sterilization methods, including autoclaving, dry heat, EtO, ethanol immersion, and antibiotic-antimycotic treatments, were shown to have only a limited impact on scaffold morphology, topography, crystallinity, and short-term cell viability. However, most sterilization techniques, except for dry autoclaving, significantly reduced the mechanical strength of the scaffolds by approximately 50%. Rnjak-Kovacina et al. (2015) [20] investigated the effects of sterilization on lyophilized silk fibroin sponges using both pre-sterilization of silk solutions (via filtration or autoclaving) and post-fabrication sterilization methods (autoclaving, γ-irradiation, dry heat, and EtO). Filtration through 0.22 μm filters was effective only for low-molecular weight and dilute solutions, whereas autoclaving produced stiffer sponges. Similarly, post-fabrication autoclaving increased scaffold stiffness and reduced degradation rates due to structural changes, while γ-irradiation accelerated scaffold degradation. Dry heat and EtO sterilization had minimal effects on protein structure. George et al. (2013) [21] evaluated the effects of γ-irradiation, steam sterilization, and ethanol immersion on silk fibroin films for corneal tissue engineering applications. Their findings showed that γ-irradiation preserved the films’ transparency (over 98%) and maintained mechanical properties comparable to those of human cornea. Ethanol-treated films also retained high transparency and exhibited suitable mechanical characteristics. In contrast, steam sterilization resulted in stronger and stiffer films with reduced transparency and altered surface topography.

While the effects of different sterilization methods on silk fibroin-based materials have been partially studied, most investigations have primarily focused on physically crosslinked films, sponges, and porous scaffolds, leaving a substantial gap in understanding how chemically crosslinked silk fibroin hydrogels in the hydrated state are affected following terminal sterilization. Considering that scaffold performance may be affected by post-sterilization structural rearrangements, a comprehensive evaluation of hydrogel properties is essential to support rational scaffold design and enable their clinical translation.

In this study, we systematically investigate the effects of several clinically relevant sterilization techniques on the functional properties of silk fibroin hydrogels obtained by photo- and enzymatic crosslinking. Particular attention is given to features that are critical for corneal tissue engineering, including transparency, mechanical strength, water content, and structural stability. By comparing scaffolds sterilized using different treatment modalities (EtO, autoclaving, γ-irradiation, and H_2_O_2_ plasma) and silk fibroin concentrations, this work aims to identify sterilization approaches that preserve or potentially enhance the desired properties of silk fibroin hydrogels for ophthalmic applications. The findings of this study are expected to contribute to the development of reliable, sterile silk fibroin-based scaffolds for corneal regeneration and broader tissue engineering applications.

## 2. Materials and Methods

### 2.1. Materials

Silk cocoons were provided by Osilk Srl (Rovereto, Italy). Na_2_CO_3_ (Merck Sigma Aldrich, Darmstadt, Germany), lithium bromide (LiBr—Merck Sigma Aldrich, Darmstadt, Germany), glycidyl methacrylate (GMA—Merck Sigma Aldrich, Darmstadt, Germany), 2,4,6-trinitrobenzene sulfonic acid (TNBS—Merck Sigma Aldrich, Darmstadt, Germany), lithium phenyl-2,4,6-trimethylbenzoylphosphinate (LAP—Merck Sigma Aldrich, Darmstadt, Germany), deuterated water (D_2_O—Merch Sigma Aldrich, Darmstadt, Germany), DMEM (Lonza Bioscience, Verviers, Belgium), hydrogen peroxidase (H_2_O_2_—Fluka Honeywell, Charlotte, NC, USA), and type VI horseradish peroxidase (HRP—Merck Sigma Aldrich, Darmstadt, Germany) were used as received.

### 2.2. Preparation of Methacrylated Silk Fibroin (SilMA) Solution

Silk fibroin was extracted from *B. mori* silkworm cocoons as described in the literature [9]. For the degumming process, 10 g of silkworm cocoons were cut into pieces and boiled for 1 h in 4 L of a 0.2 M Na_2_CO_3_ solution. The degummed silk fibroin was rinsed three times with ultrapure water for 20 min each time, and then the fibers were dried overnight under a fume hood at room temperature. A 20 wt.% silk fibroin solution was prepared by dissolving the fibers in a 9.3 M aqueous LiBr solution at 60 °C for 2 h. After dissolution, silk fibroin was reacted with GMA to produce SilMA, following the method outlined in our previous work [22]. Briefly, 1 mL of a 424 mM GMA solution per 4 g of silk fibroin fibers was added dropwise under stirring at 60 °C, and the reaction continued for 3 h. The SilMA solution was then dialyzed against ultrapure water during 4 days to remove LiBr and unreacted GMA. The solution was centrifuged at 9000 rpm for 20 min at 4 °C to remove the insoluble fraction. After that, the solution was concentrated through water evaporation under the fume hood. The final concentration was measured gravimetrically by evaporating an aliquot of the SilMA solution at 60 °C.

### 2.3. Preparation of Silk Fibroin Hydrogels

Silk fibroin hydrogels were prepared by combining photo- and enzymatic crosslinking of SilMA solution at concentrations of 5 and 7 wt.% [22]. To prepare silk fibroin hydrogels with dual crosslinking, the SilMA solution at different concentrations was mixed with LAP (0.02 wt.%), HRP (10 U/mL), and hydrogen peroxide (0.005% *v*/*v*). Then, 2 mL of the solution was poured into each well of 6-well plates for photopolymerization after exposure to UV light at 405 nm for 2 min. After photopolymerization, the hydrogels were incubated at 37 °C for 2 h to activate the enzymatic crosslinking of silk fibroin. After autoclaving the SilMA solution, hydrogels were prepared under aseptic conditions using pre-filtered LAP, HRP and H_2_O_2_ solutions.

### 2.4. Sterilization of Silk Fibroin Hydrogels

SilMA hydrogels were sterilized through various methods before further analyses. The sterilization techniques included autoclaving, γ-irradiation, EtO exposure and H_2_O_2_ gas plasma treatment. In addition, non-sterile hydrogels were prepared as a control and incubated at 4 °C in PBS supplemented with 0.05 wt.% sodium azide for subsequent physicochemical characterization.

EtO treatment: Well plates containing SilMA hydrogels were sealed in sterilization-grade pouches and exposed to ethylene oxide gas for 9 h at 45 °C. This was followed by 8 h of degassing at room temperature to eliminate any residual gas. The EtO treatment was performed in the facilities of Borgo Roma Hospital (Verona, Italy).

γ-irradiation: SilMA hydrogels prepared into well plates were irradiated using a Cobalt-60 source for a cumulative dose of 25 kGy, according to EN ISO 11137 standard [23] series. The irradiation treatment was performed in the facilities of Gammatom Srl. (Guanzate, Italy).

Autoclaving: The SilMA solution was placed in glass bottles and sterilized using a saturated steam cycle at 121 °C for 15 min under high pressure in a Steristeam 2 autoclave.

H_2_O_2_ gas plasma: Well plates containing the hydrogels were packed into sterilization pouches and subjected to H_2_O_2_ gas plasma using a STERRAD 100NX Sterilization System in the facilities of Borgo Roma Hospital (Verona, Italy).

After sterilization, all scaffolds were characterized and stored at 4 °C to assess the influence of storage conditions. The scaffolds were cut to the desired size using sterile razor blades and biopsy punches in preparation for further evaluation.

### 2.5. Material Characterization

#### 2.5.1. Determination of the Methacrylation Degree of SilMA

The degree of methacrylation of SilMA was determined using the TNBS assay [24]. This was performed by quantifying the free amine concentration of SilMA and comparing it to unmodified silk fibroin (SF), as GMA specifically reacts with the amine groups in silk fibroin, predominantly found in lysine residues. For this assay, both SilMA and SF solutions were diluted in a sodium bicarbonate buffer (pH 8.5) to a final concentration of 2 mg/mL. A calibration curve (R^2^ = 0.99) was created using β-alanine (MW = 89.09 Da) as a standard (Table S1), with concentrations of 0.000, 0.002, 0.004, 0.008, 0.016, and 0.032 mg/mL. TNBS was diluted in a 0.1 M sodium bicarbonate buffer (pH 8.5) to prepare a 0.02 wt.% solution. Then, 150 μL of each sample and standards were mixed with 300 μL of the TNBS solution and incubated for 2 h at 37 °C. The reaction with primary amines produced a yellow-orange product. After incubation, the samples were transferred to a 48-well plate, and absorbance was measured at 418 nm using a microplate reader (Tecan Infinite M200). All measurements were performed in triplicate. The degree of substitution (DS) was calculated as follows (Equation (1)):

(1) $$DS\;\%=\left[1-\left(\frac{amine\;concentration\;on\;SilMA}{amine\;concentration\;on\;SF}\right)\right]\times 100$$

#### 2.5.2. Size Exclusion Chromatography (SEC)

Molecular weight measurements were carried out using High-Performance Liquid Chromatography (HPLC) with a Shimadzu LC Nexera system. Separation was achieved using a SB-805 HQ column, specifically designed for size exclusion chromatography. Sample preparation was performed by dilution of a silk fibroin solution to a final concentration of approximately 0.2% *w*/*v* using phosphate-buffered saline (PBS) at pH 7.4, followed by filtration through 0.22 µm filters to remove particulates. For the calibration curve, two protein standard kits (Sigma-Aldrich) were employed: one with a molecular weight range of 29–700 kDa and a second kit covering a wider range of 13.7–2000 kDa. The coefficient of determination for the calibration curve was R^2^ = 0.92.

#### 2.5.3. Rheological Characterization

Rheological tests were conducted to assess the viscoelastic properties of the hydrogels using an HR2 Rheometer (TA Instruments, New Castle, DE, USA), equipped with a 25 mm stainless steel plate and a Peltier bottom plate. The rheological properties of the hydrogels were evaluated before and after the sterilization treatments using a dynamic time sweep at 1 Hz and 1% strain, continuing until the samples reached a stable modulus. To confirm linear viscoelastic behavior, dynamic frequency sweeps (1–100 rad/s at 1% strain) and strain sweeps (0.1–1000% at 1 Hz) were performed.

The viscosity of the SilMA solution before and after autoclaving was evaluated as a function of shear rate using rotational rheology with a cone-plate geometry. Measurements were performed at 25 °C over a shear-rate range of 1–300 s^−1^, and the apparent viscosity was recorded to assess the flow behavior of the precursor solutions.

#### 2.5.4. Water Content

The water content of the sterilized hydrogels was measured by incubating the biomaterials in PBS at 4 °C during 21 days. The hydrogels were weighted on days 0, 7, and 21. Before each measurement, the hydrogels were gently blotted with tissue paper to remove any excess of water on the surface, and then their weight was recorded. After that, the hydrogels were dried for 24 h at 60 °C in an oven, and the dried weight was recorded. The water content of the materials was calculated using Equation (2):

(2) $$WC\left(\%\right)=\frac{\left(W_{w}-W_{d}\right)}{W_{w}}\times 100$$

where *W_w_* is the weight of the wet hydrogel and *W_d_* is the weight of the dry sample.

#### 2.5.5. FTIR Analysis

The secondary structure of silk fibroin was examined by FTIR using a PerkinElmer Spectrum One Spectrophotometer (PerkinElmer, Waltham, MA, USA) in attenuated total reflectance mode (ATR-FTIR) in the 1300–1800 cm^−1^ frequency range, with a resolution of 4 cm^−1^ and 32 scans per spectrum. Samples were incubated in PBS medium at 4 °C and washed three times with deuterated water before measurement in order to eliminate any interference from water and the incubation medium. To assess changes in the secondary structure of silk fibroin before and after the different sterilization treatments, the amide I peak (1610–1705 cm^−1^) was analyzed using Fourier self-deconvolution followed by curve-fitting, according to an established protocol [22].

#### 2.5.6. Evaluation of the Hydrogels Transparency

The transparency of the hydrogels was monitored over time by measuring the absorbance within the 400 to 800 nm range using a Tecan Infinite M200 microplate reader. The hydrogels were incubated in PBS medium at 4 °C for a period of 0, 7 and 21 days. Absorbance values were converted to transmittance using the relation A = 2 − log10 (%T). All measurements were conducted in triplicate, with the absorbance of the PBS medium subtracted from each sample absorbance before converting to transmittance.

#### 2.5.7. Differential Scanning Calorimetry (DSC)

The thermal properties of freeze-dried SilMA hydrogels after sterilization were investigated using DSC (TA Instruments, DSC-Q20) to identify characteristic thermal transitions and degradation processes. Lyophilized silk fibroin hydrogels were subjected to a controlled heating program from 0 °C to 350 °C at a rate of 10 °C/min under a nitrogen atmosphere. The resulting DSC thermograms were compared with a non-sterile silk fibroin hydrogel.

#### 2.5.8. NMR Characterization

Solid-state NMR analyses were carried out with a Bruker 400WB spectrometer equipped with a dual band CPMAS probe. ^1^H and ^13^C NMR spectra were acquired with single and cross polarization pulse sequences (for C 100.62 MHz, π/2 3.5 µs, 2 ms contact time, 3 s recycle delay, 2k scans; for H 400.13 MHz, π/2 3.2 µs, 5 s recycle delay, 32 scans). Kinetic studies were done with variable contact time cross polarization and variable spin-lock time [25]. Samples were packed in 4 mm zirconia rotors, which were spun at 7 kHz under air flow. Adamantane CH_2_ signal at 38.5 ppm was used as external secondary reference. The line-shape fitting was performed with Bruker Topspin 3.6 software, and it was considered acceptable at 95% confidence level.

#### 2.5.9. SEM Observations

The porous structure of the hydrogels before and after sterilization was examined using a Supra 40 Field-Emission Scanning Electron Microscope (Zeiss, Oberkochen, Germany). Following sterilization, the samples were rinsed three times during 20 min with ultrapure water, and then frozen at −80 °C for 24 h. They were subsequently lyophilized at −50 °C and 0 bar for 48 h to completely remove water. Before imaging, each sample was coated with a thin conductive layer of platinum-palladium (Pt/Pd, 80:20) using a Q150T ES sputter coater (Quorum Technologies, Laughton, East Sussex, UK).

#### 2.5.10. Microbial Growth Testing

Hydrogel samples (10 mm in diameter and 2 mm in height) were sterilized by various methods and immersed into 2 mL of DMEM medium without phenol red and antibiotics under sterile conditions. The samples were then incubated at 37 °C for 7 days [26]. Following incubation, 200 µL of the medium from each sample was collected and transferred into 48-well plates, and the optical density at 600 nm was measured using a Tecan Infinite M200 microplate reader. For each condition, *n* = 3 hydrogel samples were incubated in DMEM under sterile conditions. Sterile DMEM without hydrogel served as a negative control, while a non-sterile sample was used as a positive control.

### 2.6. Statistical Analysis

The statistical analysis was conducted using GraphPad Prism version 9.0. Data are presented as mean ± standard deviation. Statistical analyses were performed using two-way analysis of variance (ANOVA) followed by Tukey’s multiple comparison post hoc test. *p*-values less than * 0.05, ** 0.01, *** 0.001, and **** 0.0001 were considered statistically significant across all analyses. Unless otherwise stated, experiments were performed using three independent samples per group (*n* = 3).

## 3. Results and Discussion

**Figure 1:**
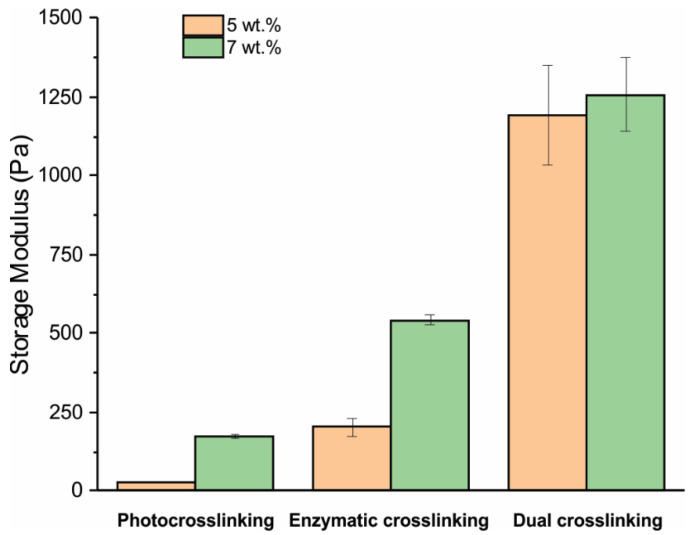
Storage modulus (G′) of silk fibroin hydrogels prepared using different crosslinking strategies (photo-, enzymatic, and dual crosslinking) at 5 and 7 wt.% concentrations.

The preparation of silk fibroin hydrogels was investigated to achieve high transparency, mechanical stability, and structural integrity required for ophthalmic applications. A degumming time of 1 h was selected to limit excessive molecular weight reduction while preserving mechanical performance and enhancing transparency of silk fibroin, as degumming conditions are known to affect chain length and, consequently, material properties [9,11]. Different crosslinking approaches were then explored for network formation, including photo- and enzymatic crosslinking strategies. Following degumming, silk fibroin was functionalized with methacrylate groups to enable controlled photopolymerization. The degree of functionalization was quantified using the TNBS assay, achieving a methacrylation degree of 89 ± 2%. Preliminary studies revealed that soft hydrogels were obtained following photocrosslinking, with storage moduli of 26.1 ± 0.8 and 174.3 ± 7.5 Pa for the 5 and 7 wt.% SilMA hydrogels, respectively, indicating low mechanical stiffness. This behavior is attributed to the inherently low content of lysine groups in silk fibroin susceptible to functionalization (approximately 0.3 mol%) [27], which limits the density of crosslinkable sites. In addition, polymer molecular weight plays a critical role in determining the final materials properties, as lower molecular weights result in reduced chain entanglement and shorter chain lengths. After 1 h of boiling and subsequent functionalization, the weight-average molecular weight of silk fibroin determined by SEC analysis was 149 kDa. For this reason, enzymatic crosslinking was also investigated to improve the mechanical performance of the silk fibroin hydrogels. The enzymatically crosslinked hydrogels yielded storage moduli values of 204.1 ± 28.4 and 542.6 ± 14.4 Pa for the 5 and 7 wt.% formulations, respectively. This increase is attributed to covalent crosslinking of the phenolic groups of tyrosine residues, which constitute approximately 5% of amino acids in silk fibroin [28]. As expected, increasing the silk fibroin concentration from 5% to 7% further enhance the storage modulus. Moreover, dual crosslinking, combining photo- and enzymatic mechanisms, resulted in a synergistic effect, significantly improving the mechanical properties. This enhancement is associated with the rapid initial network formation induced by photopolymerization, followed by further stabilization through enzymatic crosslinking [22]. The storage modulus of the hydrogels with dual crosslinking reached approximately 1190.9 ± 156.7 and 1257.8 ± 117.6 Pa for the 5 and 7 wt.% silk fibroin hydrogels, respectively (Figure 1). Dual-crosslinked silk fibroin hydrogels at concentrations of 5 wt.% and 7 wt.% were subsequently selected to evaluate the effects of different sterilization methods on the properties of materials intended for corneal regeneration.

**Figure 2:**
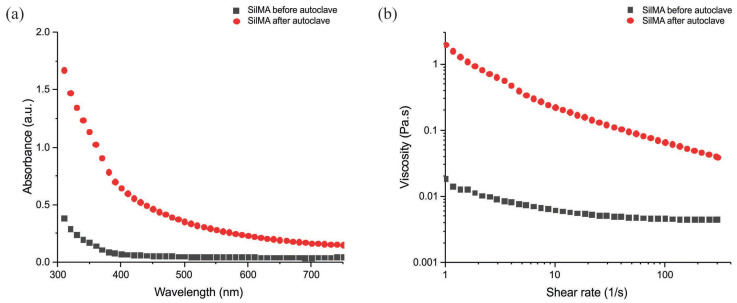
Effects of autoclave sterilization on SilMA solution: (**a**) Absorbance spectra; (**b**) Viscosity measurements.

When autoclaved, hydrogels were found to degrade under standard conditions: 121 °C for 15 min in a high-pressure saturated steam cycle. This limitation was overcome by autoclaving the SilMA precursor solution and subsequently preparing the hydrogels under sterile conditions. However, the SilMA solution showed reduced transparency and increased viscosity (Figure 2) after autoclaving, accompanied by the formation of small aggregates. Similar behavior has previously been reported for autoclaved silk fibroin solutions [20]. Representative photographs of hydrogels prepared from the untreated (control) and autoclaved SilMA precursor solutions are provided in the SI (Figure S1), illustrating the reduced transparency due to protein rearrangements and the presence of small aggregates in the autoclaved group. Autoclaving SilMA solutions alters the secondary structure of silk fibroin by promoting the formation of crystalline β-sheets, and may additionally induce premature crosslinking of the methacrylate groups While EtO and γ-irradiation serve as terminal sterilization approaches because they are performed on the fully formed SilMA hydrogels, autoclaving acts as a pre-fabrication sterilization method since it was applied to the precursor solution prior to hydrogel formation.

Furthermore, H_2_O_2_ plasma sterilization was unsuccessful as the sterilization cycle was aborted, likely due to the high water content of the silk fibroin hydrogels, which released water vapor under vacuum and interfered with stable plasma generation. As confirmed by the microbial growth testing, plasma-treated hydrogels showed detectable microbial contamination, and therefore their analysis was not considered in further discussion. Plasma sterilization was not considered a successful sterilization method under the conditions investigated and is discussed herein primarily to highlight the limitations of this approach for highly hydrated SilMA hydrogels.

**Figure 3:**
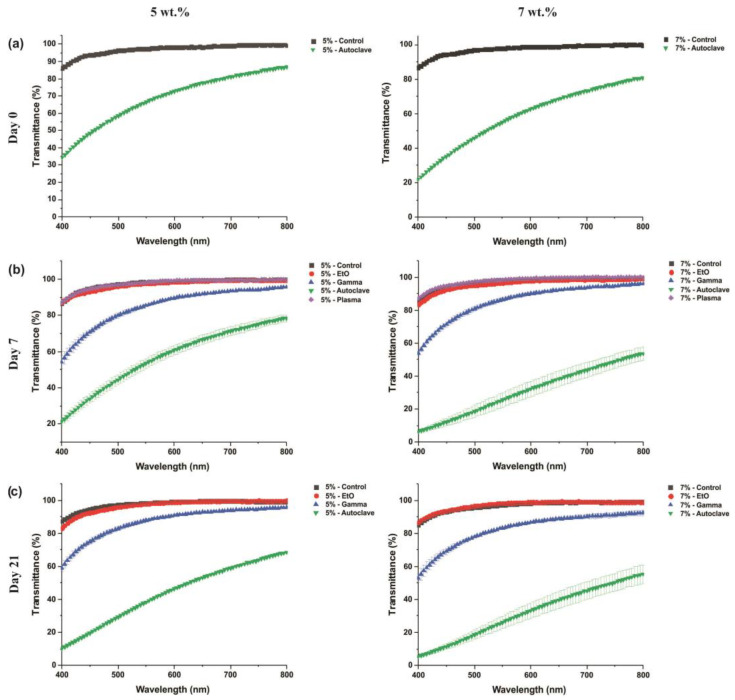
Transparency evaluation of silk fibroin hydrogels: (**a**) samples before sterilization (control) and hydrogels prepared with sterile SilMA solution (autoclave) at day 0; (**b**) hydrogels after sterilization treatments at day 7; and (**c**) hydrogels after sterilization treatments at day 21. Non-sterile samples (control) were included for comparison and were identical across all groups (EtO, γ-irradiation, and plasma) prior to sterilization.

The effect of different sterilization methods on the optical transparency of silk fibroin hydrogels was evaluated in comparison to the non-sterile control (Figure 3). EtO sterilization proved to be the most effective, preserving transparency values comparable to non-sterile samples (>98%) for both concentrations over 21 days. On the other hand, γ-irradiation resulted in a slight reduction in transparency, likely due to radiation-induced modifications in the secondary structure of silk fibroin. Nevertheless, the optical transmittance of these samples remained higher than that of the native human cornea (87.1 ± 2.0% at 500 nm) [21]. Autoclaving produced the most pronounced decrease in transparency, particularly in hydrogels at a concentration of 7 wt.%. This reduction can be attributed to structural changes induced during autoclaving of the SilMA solution, including enhanced β-sheet formation, as later confirmed by FTIR and NMR analyses. These changes not only decreased the initial transparency, but also promoted a progressive decline over time, suggesting ongoing crystallization processes within the protein network.

**Figure 4:**
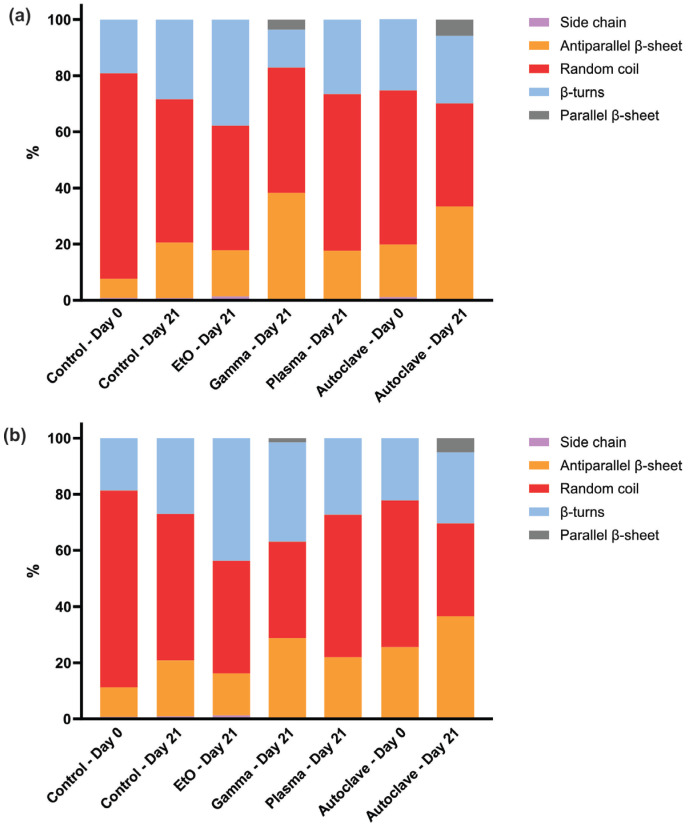
Secondary structure composition of silk fibroin determined by deconvolution of the amide I peak in the FTIR spectra of hydrogels following sterilization treatments: (**a**) 5 wt.%; (**b**) 7 wt.%. Non-sterile samples (control) were included for comparison and were identical across all groups prior to sterilization.

The secondary structure of silk fibroin hydrogels after sterilization was monitored using FTIR analysis. Figure S2 shows the infrared spectra in the 1800–1300 cm^−1^ range for the silk fibroin hydrogels before sterilization (day 0) and after sterilization. The bands observed between 1500–1700 cm^−1^ are associated with the peptide backbone, specifically the amide I (1600–1700 cm^−1^) and amide II (1500–1600 cm^−1^) absorption regions. The amide I region is particularly informative for secondary structure analysis, as it corresponds to the C=O stretching vibrations [29]. This peak encompasses contributions from various secondary structures, including side chains (1597–1609 cm^−1^), intermolecular antiparallel β-sheets (1610–1625 cm^−1^), native β-sheets (1626–1635 cm^−1^), random coils (1636–1655 cm^−1^), α-helices (1656–1662 cm^−1^), β-turns (1663–1696 cm^−1^), and intermolecular parallel β-sheets (1697–1703 cm^−1^) [30]. After hydrogel preparation, the secondary structure of silk fibroin consists predominantly of random coils, corresponding to the silk I conformation. Following incubation, the main peak in the amide I region exhibited a shoulder at 1625 cm^−1^, indicating a transition from random coils to intermolecular β-sheet crystals, which represents the thermodynamically stable form of silk fibroin. The increase in peak intensity at 1625 cm^−1^ was more noticeable in the samples sterilized by γ-irradiation and autoclaving, and at the higher SilMA concentration. The secondary structure in the amide I region was further analyzed by peak deconvolution, and the time-dependent secondary structure composition is shown in Figure 4. Sterilization treatments induced distinct structural effects. EtO caused a moderate increase in β-turn content after 21 days compared with non-treated samples, suggesting limited conformational rearrangement [17]. γ-irradiation induced more pronounced modifications, decreasing the random coil fraction and promoting antiparallel β-sheet formation, indicative of increased crystallinity. In contrast, autoclave sterilization led to immediate and significant conformational reorganization. Hydrogels prepared with autoclaved SilMA solution exhibited structural features at day 0 similar to those of untreated samples after 21 days of storage. The appearance of a strong band at 1625 cm^−1^ following autoclaving confirmed the transition to intermolecular β-sheet crystals, in agreement with previous reports showing that autoclaving increases β-sheet content and crystal size [19,27,31].

**Figure 5:**
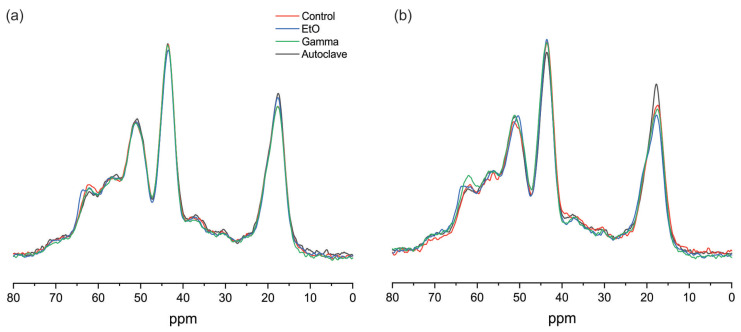
^13^C CPMAS NMR spectra of the aliphatic region of silk fibroin hydrogels after sterilization: (**a**) 5 wt.%; (**b**) 7 wt.%. Non-sterile samples (control) were included for comparison.

The structural features of silk fibroin hydrogels after sterilization were further analyzed by solid-state NMR. The hydrogel samples were lyophilized after sterilization treatments for applying the established protocol. The ^13^C CPMAS NMR spectra of the aliphatic region for all the samples are shown in Figure 5. The spectra appear superimposable, indicating that sterilization does not substantially alter the silk fibroin fingerprint. Nonetheless, minor variations in polarization efficiency (peak intensity and shape) highlight subtle dynamic differences between samples. Slight lineshape modifications are evident at the expense of the Cβ Ser peak: in EtO-sterilized samples, an additional component emerges downfield at 63.7 ppm. This resonance can be attributed to CH_2_–O carbons, arising either from residual ethylene oxide or from localized oxidation processes. Residual EtO is an important consideration for biomedical applications. Previous studies on silk fibroin biomaterials have reported that residual EtO can be substantially reduced through appropriate post-sterilization aeration and washing procedures prior to biological evaluation, resulting in acceptable cell compatibility [20]. Further studies should include dedicated residual EtO analysis and biological safety assessments, including cytotoxicity testing, to confirm the suitability of EtO-sterilized SilMA hydrogels for clinical applications.

According to the literature, the Ala Cβ resonance serves as a conformational marker (β-sheet Silk II, Silk I, random coil), as it is sensitive to secondary-structure effects due to its position along the peptide chain. Deconvolution into components at approximately 15, 17, 20, and 22 ppm—assigned to helix-like and helix/random coil (Silk I), β-sheet, and β-sheet-like (Silk II) conformations, respectively—enables the separation of structural motifs, as discussed in details elsewhere [32,33]. The corresponding profile-fitting analysis was performed following the guidelines described by Callone et al. [32]. The results of this fitting are summarized in Table S2. At 5 wt.% concentration, the non-sterile sample (control) exhibited a relatively low Silk II content (26.5%), with most of the signal corresponding to random coil/helix conformations (71.7%). Sterilization induced a pronounced increase in Silk II fractions, reaching 31.6% after EtO treatment, 37.3% following γ-irradiation, and 39.3% after autoclaving. These findings indicate that sterilization promotes structural ordering, with γ-irradiation and autoclaving being particularly effective. At 7 wt.% concentration, the control sample already displayed a higher Silk II content (34.8%) compared to its 5 wt.% counterpart, suggesting that increased concentration favors β-sheet formation. Sterilization further enhanced Silk II levels, yielding 42.2% after EtO treatment, 39.3% after autoclaving, and 49.3% after γ-irradiation. Deconvolution of Silk II subcomponents revealed that γ-irradiation and autoclaving increased both β-sheet-like (δ = 21.5 ppm) and β-sheet (δ = 20.0 ppm) contributions at the expense of random coil signals, whereas EtO treatment induced a more moderate redistribution. In agreement with FTIR results, these achievements demonstrate that sterilization not only enhances Silk II content, but also shifts the structural equilibrium toward more ordered β-sheet conformations. The increase in Silk II content is of particular relevance, as β-sheet enrichment correlates with greater crystallinity and mechanical stability of silk fibroin. These findings highlight the significant role of sterilization method and protein concentration in tuning the structural properties of silk-based biomaterials. As reported in Supplementary Materials (Figure S3), the molecular dynamics of the functional groups were also analyzed through NMR. The data both strengthen the difference among the 5 wt.% and 7 wt.% samples by highlighting an overall different crosslinking degree, and point out a slight different behavior of SilMA 7 wt.% autoclaved sample.

For the sake of completeness, the proton MAS NMR spectra were also evaluated. Figure S4 presents the spectra of the 5 wt.% and 7 wt.% hydrogel series. All spectra are characterized by a broad resonance, attributable to strong H–H dipolar couplings in the solid state. This peak arises from the superposition of all protons of the SilMA chains together with OH groups associated with adsorbed water. The right shoulder, appearing at approximately 1 ppm, corresponds to aliphatic protons and can reasonably be assumed to remain unchanged in intensity. The main resonance at 4.2 ppm can be assigned to hydrogen-bonded water protons, whereas the sharp, minor peak at 5.6 ppm is attributed to physisorbed water (“free OH”) [34,35]. For the 5 wt.% series, the OH band intensity increases along with the sequence control < γ-irradiated < autoclaved < EtO-treated, indicating progressively enhanced water retention. These results are consistent with the water content analysis of the hydrogels, as discussed later. The different retained water probably influences the local mobility of the biopolymer chain, explaining the slight different dynamics, as discussed in Supplementary Materials (Figures S3 and S5). Furthermore, an upfield shift of the free OH resonance from 5.6 to 5.3 ppm is observed in the γ-irradiated and autoclaved samples. In the EtO sample, two distinct peaks at 5.5 and 5.3 ppm are visible. Additional signals at 3.6–3.7 ppm in the EtO-treated samples suggest the presence of foreign species, consistent with residual ethylene oxide. In the 7 wt.% SilMA series, the free OH resonance is less well resolved, while the OH band increases in the order control = γ-irradiated < EtO-treated < autoclaved. The two minor peaks at 3.6 and 3.7 ppm are likewise observed in the 7 wt.% SilMA samples.

**Figure 6:**
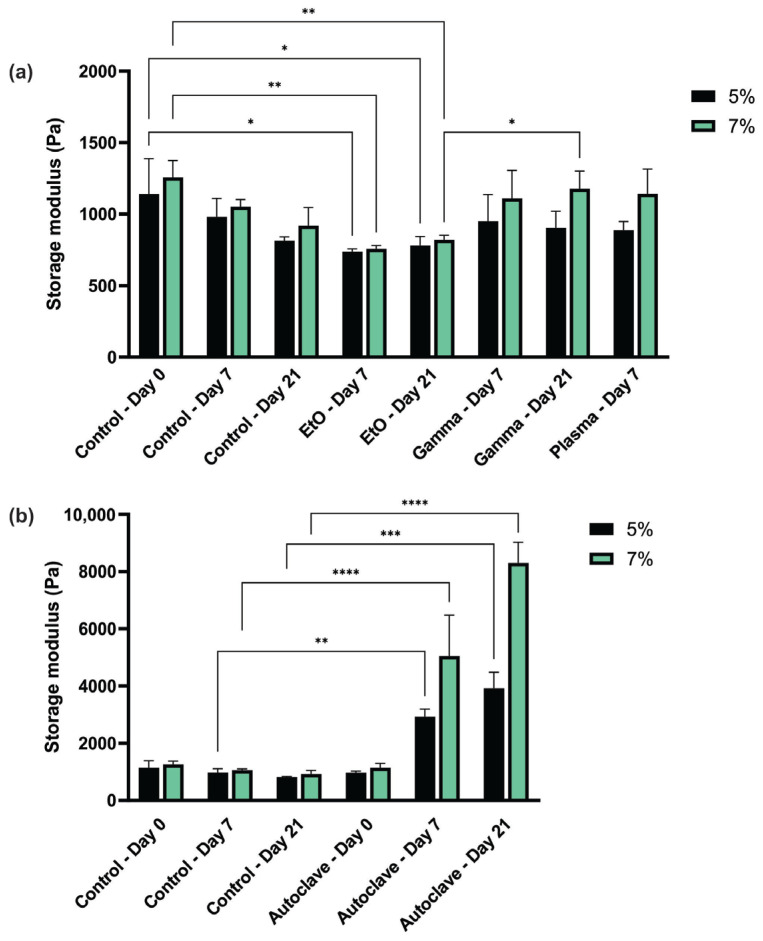
Rheological evaluation of silk fibroin hydrogels after sterilization by different methods: (**a**) EtO, γ-irradiation, and plasma sterilization methods; (**b**) Autoclave sterilization. Non-sterile samples (control) were included for comparison. The control samples on day 0, after preparation, were identical across all groups prior to sterilization.

The mechanical properties of the silk fibroin hydrogels were influenced by the sterilization method, reflecting protein degradation and secondary structure rearrangements (Figure 6). Representative strain sweep and frequency sweep data used to establish the linear viscoelastic testing conditions are provided in the Supporting Information (Figure S6). EtO sterilization led to a decrease in storage modulus, consistent with silk fibroin degradation and molecular weight decrease, which in turn decreases chain entanglements. This mechanical response is also in agreement with the higher water content, as discussed later, and the lower β-sheet formation detected by FTIR analysis. Following autoclave sterilization, the hydrogels exhibited similar rheological properties comparable to the control samples after preparation. SEC analysis showed that autoclaving the precursor protein solution reduced the molecular weight of silk fibroin from 149 to 120 kDa. Although autoclave treatment decreased molecular weight after autoclave treatment, this effect was counterbalanced by an increase in β-sheet formation, as confirmed by FTIR analysis, resulting in preserved mechanical performance. Moreover, a significant increase in the storage modulus of the biomaterials was observed after 7 and 21 days, correlating with extensive β-sheet domains formation. The increase in β-sheet content promotes physical crosslinking and reinforces the hydrogel network, resulting in stiffer materials over time. γ-irradiation preserved or enhanced mechanical stiffness, particularly at higher SilMA concentration, in agreement with the increased β-sheet content detected by FTIR and NMR analyses. Moreover, γ-irradiation could promote additional crosslinking mechanisms beyond β-sheet formation [36]. While rheological analysis demonstrated differences in viscoelastic behavior among the sterilization methods, additional mechanical studies, including compression, tensile, and stress-relaxation testing, will be required to fully assess the suitability of these hydrogels for tissue engineering applications, particularly for corneal regeneration.

**Figure 7:**
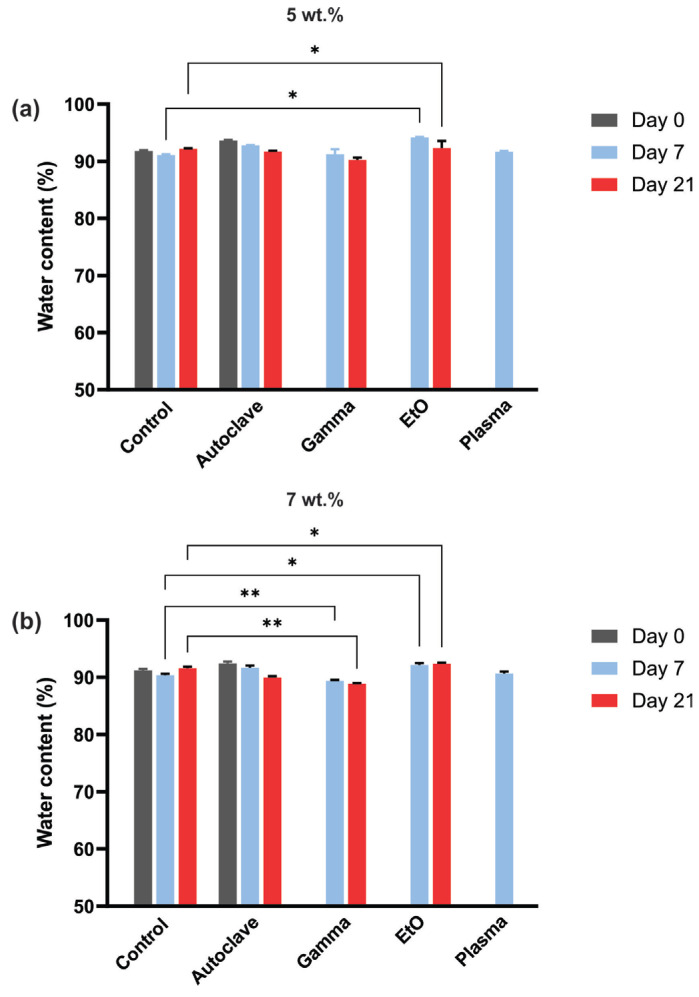
Water content of sterilized silk fibroin scaffolds using various sterilization methods: (**a**) 5 wt.%; (**b**) 7 wt.%. Non-sterile samples (control) were included for comparison. The control samples after preparation (day 0) were identical across groups (EtO, γ-irradiation and plasma) prior to sterilization.

The water content of the hydrogels was monitored after hydrogel preparation and following sterilization treatments (Figure 7). For the non-sterile hydrogels, the water content remains almost constant over time, at approximately 92 and 91% for the 5 and 7 wt.% concentrations, respectively. Both EtO and autoclaving treatments resulted in an increase in water content, with the effect being particularly pronounced after EtO sterilization, where a significant increase in water uptake was observed for both hydrogel concentrations. This behavior can be attributed to polymer chain scission during EtO treatment, leading to a reduction in molecular weight, decreased chain entanglements, and enhanced chain mobility, which collectively increase water absorption capacity. This interpretation is supported by rheological and proton MAS NMR analyses, which revealed a decrease in storage modulus and an increase in the OH band intensity after EtO sterilization, respectively. Autoclave sterilization showed a similar, but less pronounced trend, inducing only a slight and not statistically significant increase in water content compared to the control. Although autoclave treatment induces a decrease in silk fibroin molecular weight, as evidenced by SEC analysis, the concurrent increase in β-sheet content counterbalances the water absorption capacity of the hydrogels. In contrast, γ-irradiation leads to a reduction in the water retention capacity. Structural analyses by FTIR and NMR revealed an increased β-sheet content after γ-irradiation, indicating enhanced formation of ordered crystalline domains. These β-sheet structures hinder water penetration into the hydrogel network, resulting in reduced water content. This effect was statistically significant only for the 7 wt.% hydrogel, likely due to the higher initial β-sheet content and greater susceptibility to radiation-induced structural rearrangements.

The thermal properties of the hydrogels after sterilization were studied by DSC analysis. The thermograms shown in Figure S7a,b reveal the presence of three main thermal transitions in all hydrogel samples: (i) water evaporation up to approximately 150 °C, (ii) the glass transition (Tg) occurring between 150–200 °C, (iii) the onset of thermal degradation at around 250 °C. Similar thermal behavior has been reported for silk fibroin films and nanoparticles [37,38]. The thermal transitions identified from the DSC thermograms included the water evaporation temperature (Tw), glass transition temperature (Tg), and degradation temperature (Td), and the corresponding values for the 5 and 7 wt.% silk fibroin hydrogels are summarized in Table 1. Both EtO sterilization and autoclaving resulted in a decrease in Tg, indicating increased polymer chain mobility in the amorphous phase, which is consistent with protein degradation induced by these sterilization methods. These findings are in agreement with the rheological, water content and SEC results. Moreover, the degradation temperature of the hydrogels increased following sterilization compared to the control samples. This thermal stabilization is likely associated with an enhancement of β-sheet crystallinity, as β-sheet structures are known to impart increased thermal and mechanical stability due to their highly ordered arrangement [39]. With respect to γ-irradiation, a slight increase in Tg was observed post-irradiation for the 7 wt.% silk fibroin hydrogel. This increase can be correlated with the higher β-sheet content, which restricts molecular mobility within the protein matrix, as well as the formation of additional crosslinking bonds within the silk fibroin network.

**Figure 8:**
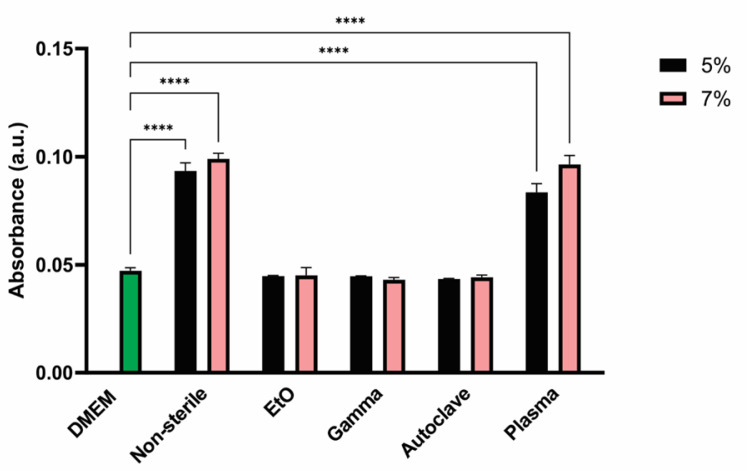
Assessment of microbial growth in culture medium and OD600 measurement. Sterile DMEM and non-sterilized hydrogels were used as negative and positive controls, respectively.

The effectiveness of the sterilization methods was assessed by incubating the scaffolds in culture medium for 7 days and measuring absorbance at 600 nm to detect bacterial growth (Figure 8). No detectable microbial growth was observed for the EtO, γ-irradiated, or autoclaved-treated samples under the conditions tested, whereas the non-sterilized control and the hydrogels treated by plasma exhibited clear evidence of microbial proliferation. This outcome is attributed to the abortion of the plasma sterilization, likely caused by the high water content of the hydrogels. Soluble compounds released from the hydrogels may potentially affect optical density measurements. However, no indication of such interference was observed under the experimental conditions employed, as the assay consistently differentiated the positive and negative controls from the sterilized samples. Although a 25 kGy dose of γ-irradiation is the standard for sterilizing medical devices, substantial research has been focused on identifying lower doses that maintain sterility while minimizing adverse effects on material properties.

**Figure 9:**
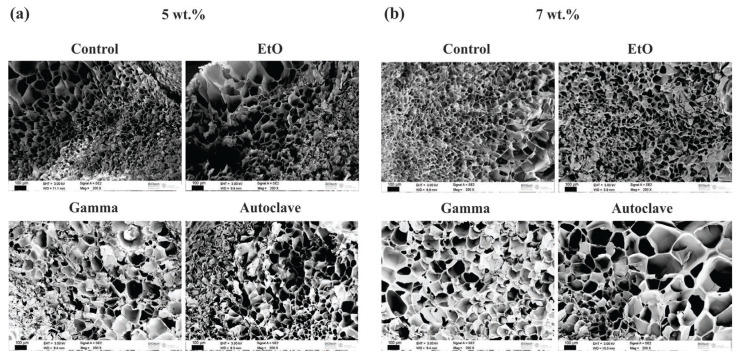
SEM micrographs at 200× magnification of non-sterile silk fibroin hydrogels (control) and hydrogels after the different sterilization methods: (**a**) 5 wt.% and (**b**) 7 wt.%. Scale bar: 100 µm.

The morphology of the silk fibroin hydrogels after sterilization was evaluated by SEM. As shown in Figure 9, all hydrogels exhibited relatively uniform macroporous structures with interconnected three-dimensional network. Regarding silk fibroin concentration, the 7 wt.% concentration showed smaller pores sizes and a more homogeneous pore size distribution, attributable to their higher crosslinking degree. Hydrogels prepared from autoclaved SilMA solution displayed larger pore size and greater heterogeneity compared to the non-sterile hydrogels, in particular for the 7 wt.% concentration. This behavior is attributed to the increased β-sheet content and viscosity of the silk fibroin solution after autoclaving, as well as the presence of aggregates, which promote the formation of pore heterogeneities. The morphology of the biomaterials was not significantly affected by the different sterilization methods [17]. However, small holes were observed in the hydrogel after γ-irradiation (Figure S8), indicating that the exposure to γ rays may induce localized material degradation.

## 4. Conclusions

This study evaluates the effects of different sterilization methods—EtO, γ-irradiation, autoclaving, and H_2_O_2_ gas plasma—on the physicochemical properties of dual-crosslinked silk fibroin hydrogels intended for ophthalmological applications. EtO and γ-irradiation were assessed as terminal sterilization methods applied to the final hydrogel, whereas the autoclave condition represents sterilization of the SilMA precursor solution prior to hydrogel fabrication. The results demonstrate that sterilization significantly influences the hydrogel transparency, mechanical performance, water content, and secondary structure of silk fibroin. Among the methods tested, EtO sterilization most effectively preserved optical transparency, making it the most suitable option for applications requiring high optical clarity. In contrast, autoclaving induced sensible structural changes in both the SilMA solution and the resulting hydrogels, leading to reduced transparency, but enhancing mechanical stability due to increased β-sheet formation. γ-irradiation also promoted β-sheet formation, potentially improving structural stability at the expense of minor optical losses. Plasma sterilization was found unsuitable due to its incompatibility with high water content, resulting in incomplete sterilization cycles and detectable microbial contamination. The microbial growth assessment demonstrated the absence of detectable microbial proliferation for the EtO-, γ-irradiated-, and autoclave-treated samples. The present study focused exclusively on the physicochemical characterization of sterilized SilMA hydrogels. Future investigations will assess the cytocompatibility and corneal cell responses of the most promising sterilization methods identified in this work, particularly EtO and γ-irradiation, to further evaluate their potential for corneal tissue engineering applications. Overall, these findings highlight the importance of selecting sterilization protocols tailored to the intended clinical application and provide a foundation for the development of sterile, structurally stable silk-based hydrogels for tissue engineering and regenerative medicine.

## Figures and Tables

**Table 1:** Thermal properties of silk fibroin hydrogels (5 and 7 wt.%) after sterilization obtained from DSC measurements.

Sample	T_w_ (°C)	T_g_ (°C)	T_d_ (°C)
5%—Control	82.4	179.1	280.9
5%—EtO	72.3	163.7	283.9
5%—Gamma	91.3	179.1	280.3
5%—Autoclave	81.7	176.4	282.8
5%—Plasma	85.1	178.0	280.1
7%—Control	86.3	178.1	280.5
7%—EtO	97.1	164.6	284.2
7%—Gamma	90.4	180.2	277.8
7%—Autoclave	85.7	176.6	285.6
7%—Plasma	91.7	177.2	280.4

## Data Availability

The original contributions presented in this study are included in the article/Supplementary Material. Further inquiries can be directed to the corresponding author.

## Supplementary Materials

The following supporting information can be downloaded at: https://www.mdpi.com/article/10.3390/polym18131625/s1, Table S1. Calibration curve with known concentration of β-alanine for the TNBS assay; Figure S1. Representative photographs of SilMA hydrogels prepared from the untreated precursor solution (a, Control) and from the autoclaved SilMA precursor solution (b); Figure S2. FTIR spectra of silk fibroin hydrogels after sterilization: (a) Day 0; (b) Day 21, 5 wt.% SF; (c) Day 21, 7 wt.% SF. Non-sterile samples (control) were identical prior to sterilization; Table S2. ^13^C CPMAS NMR Ala Cβ profile fitting results; Figure S3. Comparison of CP trends of the main functional groups in 7 wt.% SilMA hydrogels. Data from 5 wt.% SilMA hydrogels are included for reference; Figure S4. ^1^H MAS NMR spectra of silk fibroin hydrogels after sterilization: (a) 5 wt.% concentration; (b) 7 wt.% concentration. Non-sterile samples (control) were included for comparison. Spectra were normalized to the right shoulder at about 1 ppm, due to the aliphatic protons; Figure S5. ^1^H MAS T1ρH relaxation trends for the different sterilization treatments; Figure S6. Representative strain sweep and frequency sweep measurements of 5 and 7 wt.% SilMA hydrogels (control). These data were used to define the rheological testing parameters applied in the study; Figure S7. DSC thermograms of silk fibroin hydrogels after sterilization: (a) 5 wt.%; (b) 7 wt.%. Non-sterile samples were included for comparison; Figure S8. SEM micrographs of silk fibroin hydrogels after γ-irradiation sterilization at 500× magnification: (a) 5 wt.%; (b) 7 wt.%.

## Abbreviations

The following abbreviations are used in this manuscript:

ATR-FTIR	Attenuated Total Reflectance Fourier Transform Infrared Spectroscopy
CPMAS	Cross-Polarization Magic Angle Spinning
DSC	Differential Scanning Calorimetry
DMEM	Dulbecco’s Modified Eagle Medium
D_2_O	Deuterated Water
EtO	Ethylene Oxide
GMA	Glycidyl Methacrylate
H_2_O_2_	Hydrogen Peroxide
HRP	Horseradish Peroxidase
LAP	Lithium Phenyl-2,4,6-Trimethylbenzoylphosphinate
LiBr	Lithium Bromide
NMR	Nuclear Magnetic Resonance
OD	Optical Density
PBS	Phosphate-Buffered Saline
SEC	Size Exclusion Chromatography
SEM	Scanning Electron Microscopy
SilMA	Methacrylated Silk Fibroin
Tg	Glass Transition Temperature
TNBS	2,4,6-Trinitrobenzene Sulfonic Acid
γ-irradiation	Gamma Irradiation
