# Supplementary material for: Effect of Sterilization Methods on the Physicochemical Properties of Silk Fibroin Hydrogels
Source: Polymers (Basel). 2026 Jun 30;18(13):1625. doi: 10.3390/polym18131625 (PMC13363871; doi:10.3390/polym18131625)
Supplement: Supplementary file 1 [file polymers-18-01625-s001.zip › polymers-4355693-supplementary.pdf]

Supporting information

Table S1. Calibration curve with known concentration of β-alanine for the TNBS assay.

Concentration (mg/mL)	Molarity (M)	Absorbance (A)
0.002	2.245×10^{-5}	0.036
0.004	4.490×10^{-5}	0.079
0.008	8.980×10^{-5}	0.155
0.016	1.796×10^{-4}	0.322
0.032	3.592×10^{-4}	0.628

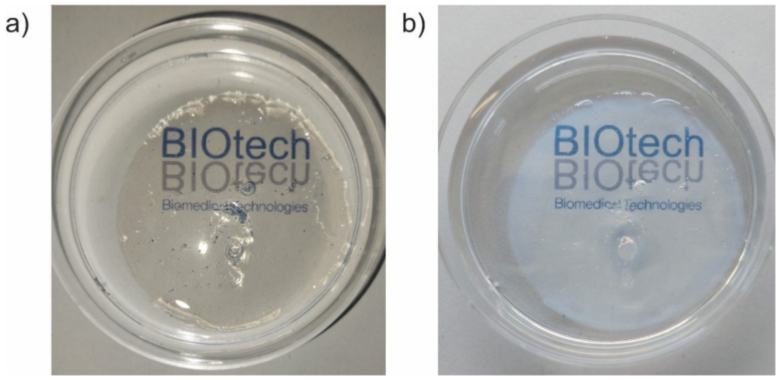

Figure S1. Representative photographs of SilMA hydrogels (5%) prepared from the untreated precursor solution (a, Control) and from the autoclaved SilMA precursor solution (b).

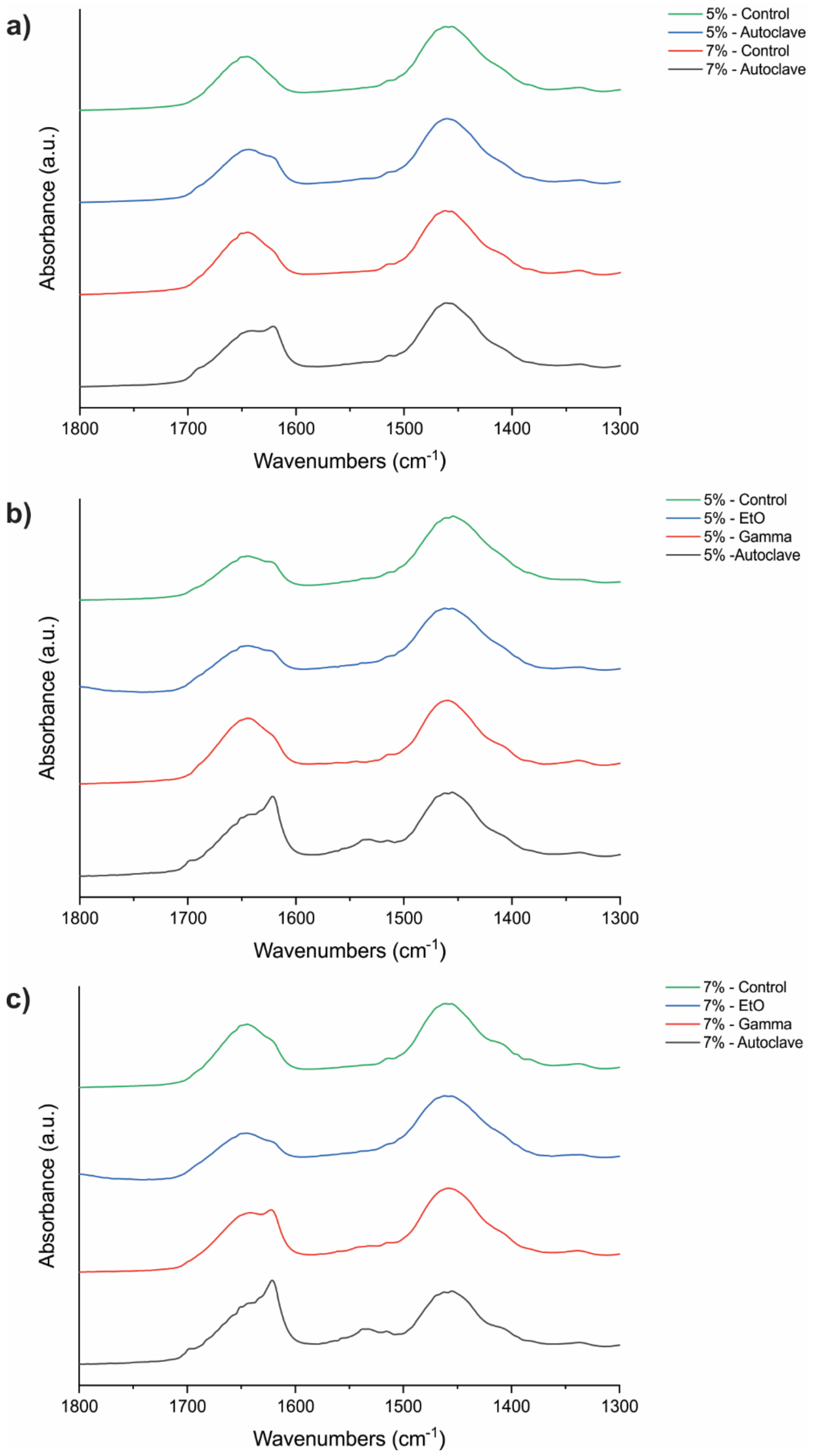

Figure S2. FTIR spectra of silk fibroin hydrogels after sterilization: a) Day 0; b) Day 21, 5 wt.% SF; c) Day 21, 7 wt.% SF. Non-sterile samples (control) were identical prior to sterilization.

Table S2. ^{13}C CPMAS NMR Ala Cβ profile fitting results.

	Silk I-like/rc		Silk II		
	helix-like	helix/random coil	β-sheet	β-sheet-like	% Silk II $_{TOT}$
δ (ppm)	16.9	17.5	20.0	21.5	
5% - Control	1.7	71.7	19.0	7.6	26.5
5% - EtO	5.6	62.8	24.4	7.2	31.6
5% - Gamma	3.8	58.8	24.3	13.0	37.3
5% - Autoclave	7.4	53.3	28.7	10.6	39.3
7% - Control	4.9	60.3	27.6	7.2	34.8
7% - EtO	2.3	55.6	29.2	13.0	42.2
7% - Gamma	0.5	50.2	34.1	15.2	49.3
7% - Autoclave	1.7	59.0	31.2	8.1	39.3

Additional insights into the dynamic behavior of the material can be obtained from solid-state NMR by analyzing the dynamics of the cross-polarization (CP) process. Since CP is promoted by dipolar I–S interactions, which are intrinsically sensitive to internuclear distances, it serves as a powerful probe of short-range ordering and local dynamics. At the initial stage of CP, the ^{13}C magnetization is polarized through ^{1}H–^{13}C heteronuclear dipolar interactions mediated by the ^{1}H reservoirs. The growth of spin magnetization at this stage is governed by the cross-polarization rate constant, T_{CH}, which depends on the relaxation behavior of both ^{1}H and ^{13}C nuclei as well as the effective strength of the dipolar interaction (determined by the C–H distance and molecular motions). At longer contact times, ^{13}C magnetization follows an exponential decay described by the proton spin–lattice relaxation time in the rotating frame, $T_{1\varrho H}$. Measurement of $T_{1\varrho H}$ at resolved carbon resonances provides information on the relative mobility of protons in the molecular framework and can reveal the presence of morphological heterogeneity. The kinetics of the Cα and CO carbons are presented in Figure S2, as representative. The displayed trends are similar among the samples, indicating that the backbone is largely unaffected by conformational differences potentially induced by sterilization.

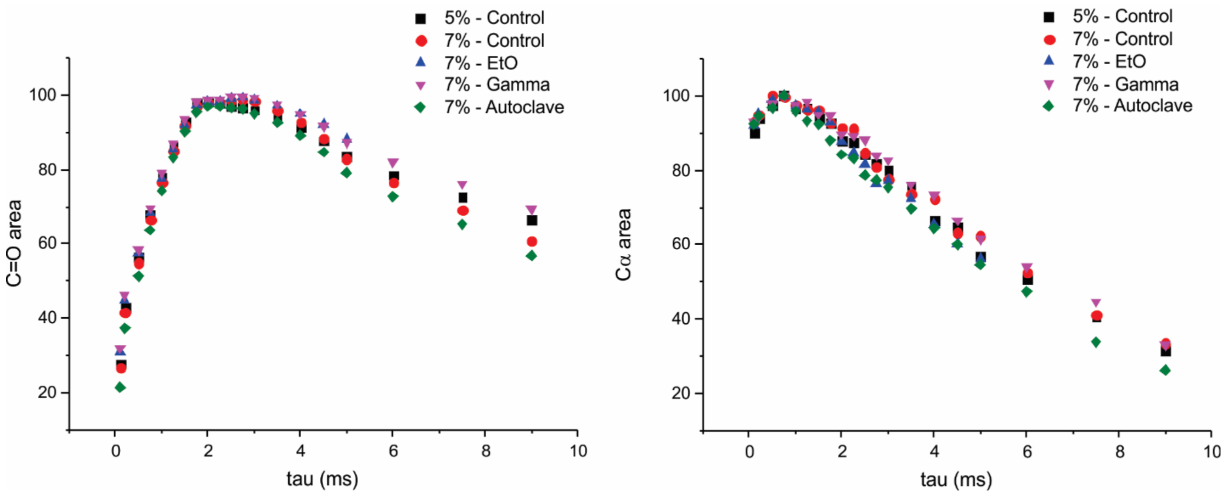

Figure S3. Comparison of CP trends of the main functional groups in 7 wt.% SilMA hydrogels. Data from 5 wt.% SilMA hydrogels are included for reference.

The 7 wt.% SilMA hydrogel sterilized by autoclaving exhibits a faster $T_{1\varrho H}$ compared to the sample sterilized by gamma irradiation, which shows a slower relaxation. This suggests that autoclaving induces slower dynamics due to more rigid or crystalline regions, while gamma sterilization promotes higher local disorder.

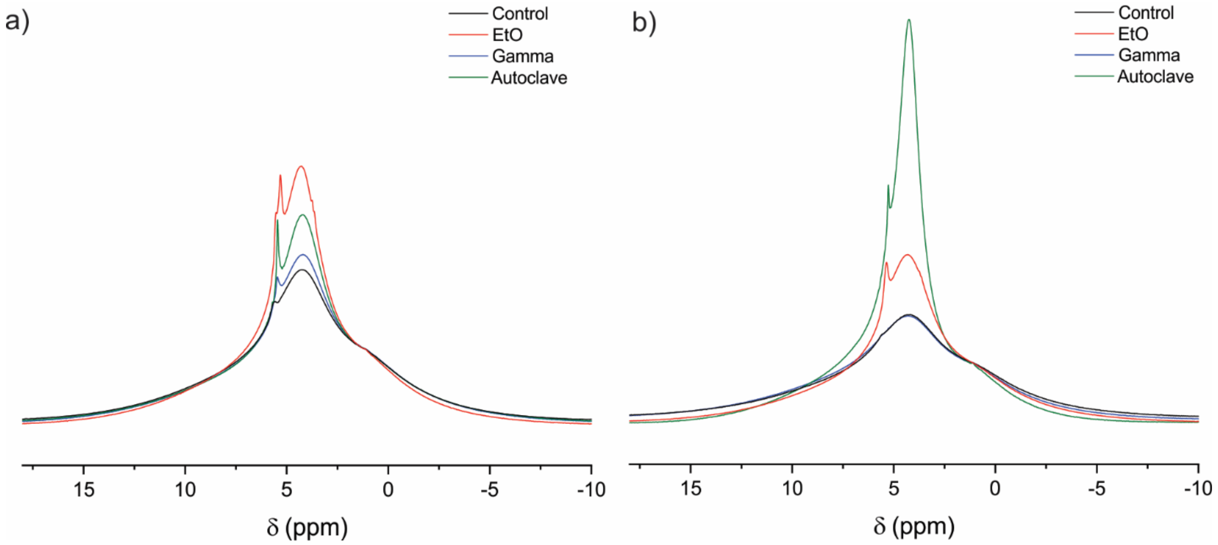

Figure S4. ^{1}H MAS NMR spectra of silk fibroin hydrogels after sterilization: a) 5 wt.% concentration; b) 7 wt.% concentration. Non-sterile samples (control) were included for comparison. Spectra were normalized to the right shoulder at about 1 ppm, due to the aliphatic protons.

The proton NMR dynamics of the material was analyzed also though a direct $T_{1\varrho H}$ measurement in ^{1}H MAS NMR. This technique allows for the study of molecular motion and local environments in solid materials, providing insights into phenomena like methyl group rotation and segmental motion in polymer chains by measuring how protons recover their magnetization after being perturbed. The decay trends are presented in Figure S4. Since multiple types of hydrogens are overlapped into the main resonance and different chain packing have been found, the trend cannot be linear and multiple relaxation times constants should be detected. The trends can be divided into three regions with different slopes: an initial fast decay, due to fast local motions and constant across samples; an intermediate decay, more variable thus sensitive to processing; and the final slow relaxation probably due to adsorbed water. The intermediate motion regimes highlight the different behavior of SilMA 7 wt.% autoclave whose molecular mobility appears clearly slower with respect to the other samples. Probably the higher amount of retained water affects the local mobility of the biopolymer chains.

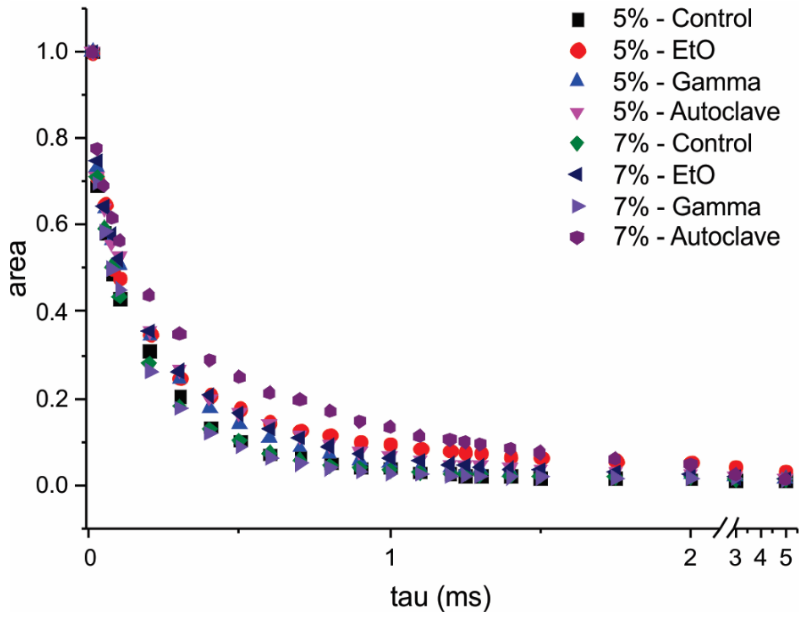

Figure S5. ^{1}H MAS $T_{1\varrho H}$ relaxation trends for the different sterilization treatments.

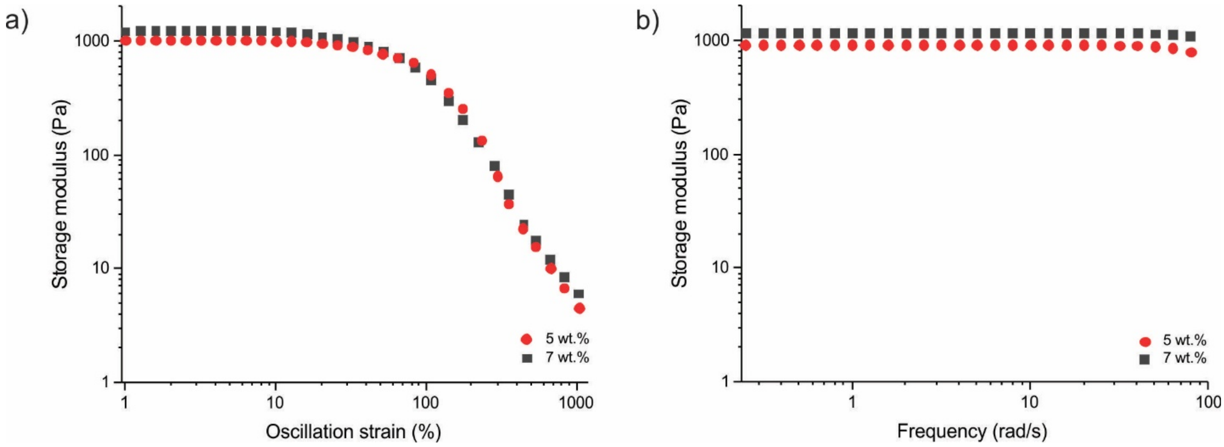

Figure S6. Representative strain sweep and frequency sweep measurements of 5 and 7 wt.% SilMA hydrogels (control). These data were used to define the rheological testing parameters applied in the study.

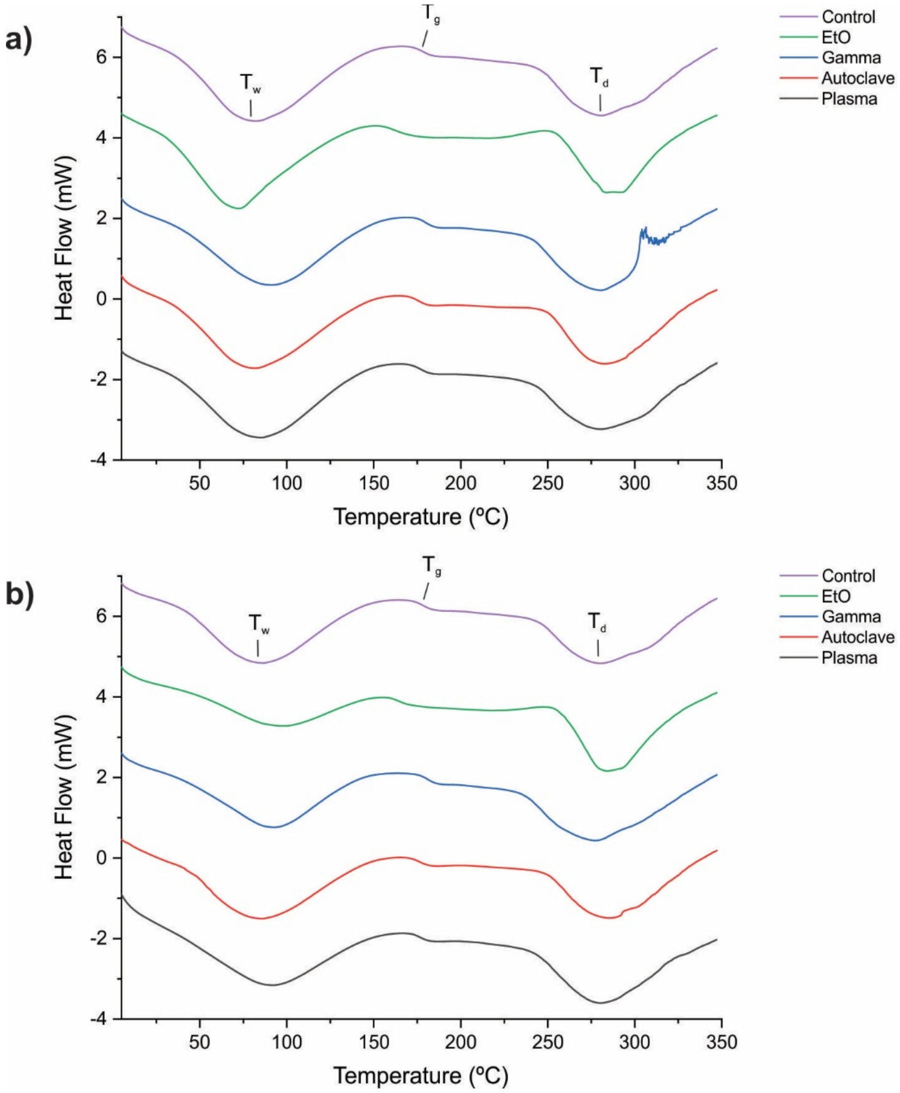

Figure S7. DSC thermograms of silk fibroin hydrogels after sterilization: a) 5 wt.%; b) 7 wt.%. Non-sterile samples were included for comparison. T_w corresponds to the water evaporation temperature, T_g to the glass transition temperature, and T_d to the degradation temperature.

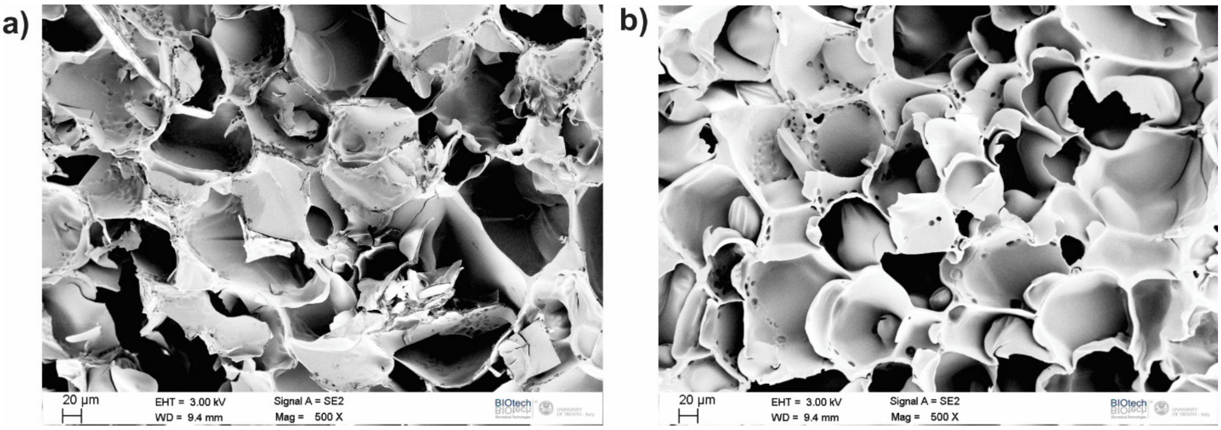

Figure S8. SEM micrographs of silk fibroin hydrogels after gamma irradiation sterilization at 500X magnification: a) 5 wt.%; b) 7 wt.%.
